# Transcranial and muscular single-pulse magnetic stimulation is efficient on motor functional neurological disorders by the feedback of induced muscle contractions — A retrospective case series

**DOI:** 10.1016/j.prdoa.2021.100112

**Published:** 2021-10-20

**Authors:** Mickael Bonnan

**Affiliations:** Service de Neurologie, Centre Hospitalier de Pau, 64000 PAU, France

**Keywords:** Conversion disorder, Motor-evoked potentials, Transcranial magnetic stimulation, Magnetic field therapy, Suggestion, Functional neurological disorder

## Abstract

**Objective:**

Motor functional neurological disorders (mFNDs) are improved by repetitive transcranial magnetic stimulation (rTMS), which is thought to involve cortical modulation. We examined the outcome of a rapid TMS procedure.

**Methods:**

Single-center retrospective case series including 41 consecutive patients suffering from mFNDs and receiving a combination of motor-evoked potentials (MEP), TMS and/or muscle stimulation.

**Results:**

MEP and additional TMS were administered in 35 patients, sometimes with rescue by muscle stimulation. Magnetic muscle stimulation was given in 6 patients, sometimes with rescue by TMS. Complete immediate recovery was obtained in 65.9 % of the 41 patients, but the outcome of mFNDs after one year was poor. Treatment by TMS (n = 19) or by muscle stimulation (n = 4) given alone were associated with 78.9 % and 75 % of complete immediate recovery, respectively.

**Conclusions:**

A rapid easy-to-perform TMS procedure obtained a high rate of immediate complete recovery in mFND. Clinical recovery was improved but was also obtained by direct magnetic stimulation of the paralyzed muscles.

**Significance:**

TMS-induced recovery of mFND may not involve cortical modulation but could rather occur through reinforcement of the suggestion. Magnetic-induced muscle twitches may facilitate the self-expectation of motor recovery and could unlock the motor symptoms of mFND.

## 1 Introduction

Motor functional neurological disorders (FNDs) are common disabling neurological symptoms that do not conform to known anatomical pathways. Although the diagnosis of FND is based on positive signs, a somatic check-up is usually required to rule out the rare presentation of clinical mimics [1]. Many pathophysiological pathways have been proposed, but none of them is suitable to cure FNDs at the bedside, so care is largely empirical [1]. Psychiatric or behavioral therapies are often proposed to cope with the associated depression or anxiety signs associated with the triggering psychological stressor, but the treatment of the underlying personality disorder remains difficult and is always delayed. Moreover, patients and their relatives often have misgivings about functional explanations as long as the impairment remains dramatic, so the rapid alleviation of symptoms in persistently impaired patients remains challenging.

Repetitive transcranial magnetic stimulation (rTMS) has demonstrated a high rate of improvement, suggesting an effect of cortical modulation on motor FNDs (reviewed in [2]. Although transcranial magnetic stimulation (TMS) is highly efficient, its mechanisms of action are unclear, and its contribution remains questionable since FNDs usually improve with supportive psychotherapy, hypnosis, placebo or even spontaneously.

Motor-evoked potentials (MEPs) can confirm that the motor pathway is spared but can also provide proof of muscle contraction to the patient, thereby leading to immediate recovery. We report a large single-center series of mFNDs immediately improved after single-pulse TMS. This successful clinical experience was also obtained by magnetic stimulation of the involved muscles, suggesting that instead of cortical modulation, magnetic-induced muscles twitches could be the main trigger that unlocks motor palsy.

## 2 Material and methods

### 2.1 Setting

All patients diagnosed with mFNDs from March 2012 to December 2019 and referred from neurological or emergency consultations at Centre Hospitalier de Pau were included. Procedures used in this study were all part of common care provided for mFND since 2011 [3]. Data including procedure and clinical outcome were prospectively recorded in the medical files, and retrospectively included in the study. All patients were first examined by a neurologist (MB) and received brain or spinal cord MRI when appropriate to rule out an organic disorder. All patients met the diagnostic criteria for FND, according to Diagnostic and Statistical Manual of Mental Disorders-5th Edition (DSM-V) diagnostic criteria. This study fulfils French law controlling non-interventional retrospective studies using de-identified data.

### 2.2 Procedures and groups

Patients were told that it was very likely that their benign disorder would be cured by TMS, if required. A second appointment was given in the neurophysiological ward within a week (see Table 1 for procedure). First, patients were told about MEP, how they are measured, and how benefits were expected. After their consent was obtained, MEPs were recorded on four limbs to confirm normal motor conduction. Normal nerve function from the brain to the distal limbs was explained throughout the test. If confirmed at the end of the procedure, the diagnosis of FND was explained by using the analogy of an electric circuit breaker. Patients were told that TMS is highly efficient to switch on motor circuitry by a complex but as yet unknown action on brain, and they were encouraged to see whether an improvement had already occurred during the MEP procedure. The latter was usually complemented by TMS sequences (C group) and followed by a clinical examination. If there was no recovery, muscle stimulations were proposed to rescue TMS (C → M group). A few patients received muscle stimulation alone (M group), and those failing to improve were given rescue TMS (M → C group). Rescue procedures were interrupted if no improvement was observed after two attempts.

**Table 1:** Flow chart of study inclusion and procedures. All procedures were done during a single consultation. Expected recovery was complete and immediate.

### 2.3 Magnetic stimulation

Magnetic stimulation was done with a single-pulse TMS using a circular coil (MagPro, MagVenture) coupled with an electromyographics device (Dantec Keypoint) to acquire MEP. MEP were acquired on both sides. Cortical representation of upper and lower limbs was identified by surface EMG of the *abductor digiti minimi* muscle of the hand or the *abductor hallucis* muscle of the foot. Intensity of TMS was set to supra-threshold intensity to obtain a reproducible EMG signal and was left unchanged during additional TMS. Intensity of muscle stimulation was adjusted to obtain a painless, clear but minimal muscle contraction (i.e. evident for the patient himself). Painless stimulation was always demonstrated on the neurologist’s forearm to reassure the patient. TMS was delivered in sequences of 10 magnetic stimulations (manual trigger) on each side of the motor cortex. Direct muscle stimulation was given in sequences of 10 stimulations delivered on the extensor and flexor muscle groups (arm and forearm, thigh and calf) of the involved limbs.

### 2.4 Analysis

Clinical results were ascertained at the end of the procedure and stratified in three classes corresponding to a simplified Clinical Global Impression-Improvement (CGI-I) score: immediate complete improvement (CGI-I 1); partial improvement (CGI-I 2 and 3); absence of apparent effect (CGI-I 4). Statistical analyses of count data were done with Fisher's exact test, and *p* value was set to 0.05. Excel (v16.16.22, Microsoft, 2018) was used for graphical results and analysis.

## 3 Results

Among 43 patients with FNDs referred for treatment with magnetic stimulation, two patients were excluded: one patient with sensory-motor (SM) hemiparesis refused the procedure, and an organic disorder (Barré syndrome) was discovered during the procedure in another. Forty-one patients were included (Supplementary Table S1, median age was 36.5 [14 to 64] years, and 87.8 % were female (Table 2. Clinical symptoms were the following: paraparesis of lower (n = 12) or upper limbs (n = 1), monoparesis of leg (n = 8), arm (n = 3), or hemiplegia (n = 1), gait disorder (n = 10), sudden falls (n = 1), functional movement disorders (n = 4), mutism (n = 1), and blindness (n = 1). Two cases of functional tremor were associated with other symptoms. Lateralized symptoms involved the right limb in 8 (64.3 %) patients. MEP gave normal results in all cases.

**Table 2:** Demographics and main results. a Proportion within non-rescued group.

	Results, % (n)
Female	87.8 (36)
Age, median [min–max]	36.5 [14 to 64]
Pediatric cases (≤16 years)	14.6 (6)
*Symptoms*	
Bilateral motor palsy Unilateral motor palsy Gait disorder Movement disorders Other	31.7 (13) 26.8 (11) 24.4 (10) 9.8 (4) 7.3 (3)
Onset within a year	85.4 (35)
*Procedure*	
TMS (C) —rescue muscle stim. (C → M) Muscle (M) —rescue TMS (M → C)	85.4 (35) 45.7^a^ (16) 14.6 (6) 33.3^a^ (2)
*Recovery*	
Complete immediate Incomplete Absent	65.9 (27) 19.5 (8) 14.6 (6)

Delay from symptom onset to procedure was within two days in 26.8 %, within 2 weeks in 53.6 %, within up to a year in 85.4 %, or more than a year in 14.6 %. Improvement was complete, incomplete or absent in 65.9 %, 19.5 % and 14.6 %, respectively. No symptoms worsened during the procedure. Delay of onset longer than a year was associated with poor recovery (16.7 % vs 74.3 %; *p* = 0.013).

**Fig. 1:**
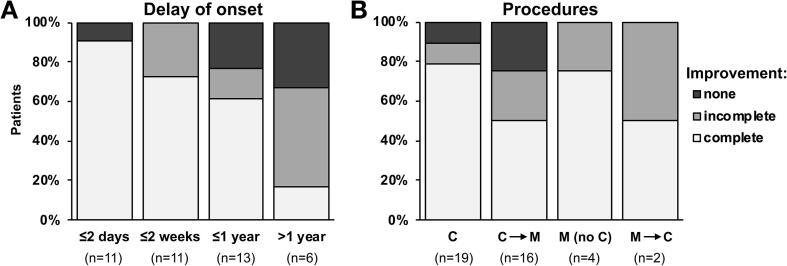
Immediate outcome of procedures (n = 41). A. Influence of delay of onset. Better outcome was obtained in patients undergoing early procedures. **B.** Influence of procedure. Complete improvement was obtained in most patients undergoing TMS, but also in patients who received muscle stimulation without TMS.

Complete improvement was obtained in 78.9 % after TMS alone (C group), and in 50 % of cases who received complement muscle stimulation (C → M group) (Fig. 1. Muscle stimulation (M group) used alone, without preceding TMS or MEP, led to complete improvement in three of four patients (75 %). Two more patients who did not improve after muscle stimulation also received TMS (M → C group) and one more improved. Complete recovery was obtained in 70.8 % of motor symptoms and 58.8 % in other FNDs (*p* = 0.51). Complete recovery was transient in one case, and one incomplete recovery at the end of the procedure became complete within days. No side effect occurred during the procedures.

## 4 Discussion

Our results are similar to previous findings concerning female predominance, young population, clinical signs, proportion of complete improvement, and influence of delay of onset on outcome [[3], [4]]. On the other hand, some findings were new. First, we found that single-pulse TMS given during MEP or repeated during a very short sequence was as efficient as rTMS [[4], [5], [6], [7], [8], [9], [10]]. The whole procedure took less than half an hour and was conducted on an inexpensive, simpler TMS device than the rTMS devices which use positioning systems to target cortical areas with precision over repeated procedures. Although the pathophysiology of FND remains unclear, TMS proved to be beneficial. Therefore, it would seem that the use of TMS in neurological treatment is not *etiology-driven* but rather *technologically-driven* [11], whereby clinicians tend to rule out the eventuality of a placebo effect and consider that neuromodulatory or neurophysiological processes are involved [[3], [4]]. However, a randomized rTMS study found that cortical modulation may not be required to obtain an effect on functional movement disorders [8]: a similar rate of improvement was obtained in patients stimulated on the contralateral cortical motor area or the homolateral spinal root. The authors proposed that, in combination with the power of suggestion, involuntary muscle twitches elicited by suprathreshold stimulation may have promoted remission. However, the rate of complete improvement was low (30 % reached CGI-I 1), possibly due to the variety of functional disorders, and all patients finally received cortical stimulation in a cross-over design. Muscle twitches elicited by spinal root stimulation in that study remained minor as compared with the powerful contractions we obtained by direct magnetic stimulation of the muscles, which constitute painless, easy-to-demonstrate proof of preserved motor function. Evidence of induced muscle contraction may explain the variability of results obtained with different TMS protocols used in the literature. Studies using cortical stimulation below or around the motor threshold mostly led to only minor improvement [[5], [12], [13], [14]], whereas those using an intensity above the threshold gave spectacular results [[3], [6], [8], [9], [10], [15], [16]].

We therefore added direct muscle stimulation as a rescue procedure after TMS in patients failing to regain immediate and complete improvement. This bias may explain the slightly lower outcome of the C → M group compared with the C group. Finally we gave muscle stimulation (M group) only to the last 6 patients and used TMS as a rescue technique in 2 patients. Overall good outcome was similar between the M and C groups, and the rescued M → C and C → M groups. This is major evidence against TMS-induced cortical modulation. We hypothesize that the evidence of movements induced by stimulation targeting either the cortex or muscles in motor-impaired patients could be both a major feedback cue and the key factor explaining the efficiency of TMS. Lastly, magnetic stimulation may seem to patients to be a high-technology procedure, thus reinforcing their naive expectation of experiencing a spectacular clinical benefit, and possibly allowing them to cast off what is, in effect, an inorganic symptom while saving face. Consequently, we propose that, while TMS could be devoid of neurophysiological activity in FND, it is far more than being a simple placebo since it objectively fulfills the self-expectation of recovery by acting as an *'active placebo'*. While more patients are needed to confirm this hypothesis, especially in groups M and M → C, the dramatic responses that we obtained suggest that this simple method efficiently cures motor FND.

Most of our patients were at least partly improved immediately after MEP, and additional or rescue TMS and/or muscle sequences were given to maximize recovery. Improvement usually became greater over a few minutes, during which patients often tried to regain movement after several attempts, increasingly succeeding until they reached complete recovery. Additional sequences of magnetic stimulations given between these attempts were also helpful. Procedures described until now in the literature have usually spanned several sessions and the recovery rate was assessed only after they had been completed. Our criterion of improvement required complete and immediate recovery (CGI-I 1) obtained at the end of the medical consultation, in order to minimize the putative effects of neuromodulation. Patients failing to improve fully during their consultation were considered as failures in this study, which lowered the success rate. They were informed that they could expect improvement subsequently, and this occurred in at least one patient in the following days. However, systematic follow-up was not available.

Improvement of motor symptoms without limb plegia such as functional gait disorders was less impressive than that of pure motor symptoms. We did not assess muscle stimulation on non-motor FND (e.g. sensory or cognitive complaints) where the feedback cue would be less appropriate, although good results were obtained [17]. We believe that the lower efficiency of TMS obtained in series of non-paralytic movement disorders such as functional tremors [[8], [9], [14]] and mutism may stem from the inconsistency of motor feedback with FND symptoms. For instance, for the feedback of tremors to be consistent, it would require a procedure able to briefly halt the tremor, which is hardly available at present. The objective muscle contraction induced by TMS using intensity above motor threshold prevents future blinded studies, and consecutive non-randomized patients were included in our different procedures. However, since complete recovery was a reachable goal, these limitations could hardly be a bias. We cannot exclude that suggestion of recovery would have been sufficient to obtain full recovery, even though none of the symptoms had vanished before the stimulation procedures were undertaken, although a high level of suggestion was attained. Other studies using strong suggestion before stimulation below the motor threshold failed to obtain such an improvement [13], and immediate recovery was not a common feature in motor FNDs treated only by hypnosis [18]. We did not collect our historical cases. Immediate complete recovery, as observed in this study, was hard to obtain by simple suggestion before the era of magnetic stimulation.

All our patients received complete paraclinical tests to rule out somatic disorders. Patients presenting with unclear motor symptoms and completely normal paraclinical exams should exhibit normal MEP to rule out the suspicion of organic disorder. One of the two patients excluded from analysis displayed abnormal MEP that revealed an acute Guillain-Barré syndrome (GBS). He had been suffering from bizarre gait with variable weakness and spared reflexes for two days. Lumbar puncture, biological tests, brain and spinal MRI and EMG including normal motor conduction, A and F waves, were all normal, and FND was tentatively diagnosed by multiple neurologists. TMS was proposed to alleviate a probable FND, but the very low amplitude of motor responses elicited by cortical stimulation led to the unexpected diagnosis of proximal motor blocks. Typical signs of GBS became apparent over the coming days but improved after intravenous IgG (IVIG) infusion. This unique organic case (2.3 %) among our series of FNDs underlines the value of checking MEP once before curing motor FND with TMS, especially in patients failing to regain normal function immediately.

MEP were done to definitively rule out pyramidal involvement in patients suffering from mFND. Although improvement following MEP was described in patients with motor symptoms (i.e. involving pyramidal pathway in limbs) [3], other symptoms were also improved (e.g. speech, vision) [[6], [17]]. Is it ethical to use the side-effects of MEP as a treatment of FND? A psychodynamic role was often given to symptoms, supporting the traditional idea of '*belle indifférence*', and underlying that persistence of disability would be desirable to the patient itself. Patients often face to hostile reaction of the medical staff undermined by the thought that the patient is malingering. In fact, patients are really disabled, seek diagnosis, demand effective treatments, and persisting FND are associated with severe disability [19]. We believe that adherence to diagnosis and outcome are improved in patients treated early after FND onset. Our patients were told about the two expected roles of MEP (diagnosis and treatment), they were interested, and this procedure was considered respectful of their expectations. They were satisfied of dramatic improvement. Although we cannot completely exclude suggestion or transference in this case series, our observations also suggested that muscle twitches triggered by diagnosis MEP might be key players.

For the management of motor FND, we propose first acquiring MEP data to obtain proof of preserved normal function. Patients failing to improve after this procedure may receive additional TMS and muscle stimulation to reinforce the evidence that motor function is preserved. Onset of FND is usually associated with life events and social triggers unmasking psychological weaknesses, which are not cured by symptom alleviation. Dramatic motor improvement offers an opportunity to the therapist to confidently broach the need for further psychological support to prevent relapses.

## 5 Conclusions

Magnetic stimulation easily and immediately alleviates motor FNDs in most patients. Apart from the strong suggestion given during the exam, our data suggest that muscle twitches induced by TMS or direct muscle stimulation play a major role in motor recovery.

## CRediT authorship contribution statement

**Bonnan Mickael:** Conceptualization, Data curation, Formal analysis, Investigation, Writing – original draft, Writing – review & editing.

## Declaration of Competing Interest

The authors declare that they have no known competing financial interests or personal relationships that could have appeared to influence the work reported in this paper.
