# Supplementary material for: Transcranial and muscular single-pulse magnetic stimulation is efficient on motor functional neurological disorders by the feedback of induced muscle contractions — A retrospective case series
Source: Clin Park Relat Disord. 2021 Oct 20;5:100112. doi: 10.1016/j.prdoa.2021.100112 (PMC8554265; doi:10.1016/j.prdoa.2021.100112)
Supplement: Supplementary data 1 [file mmc1.docx]

Supplementary Material

# Supplementary Table S1. Patient procedures and outcome

Anonymized dataset. ^a^ same patient with recurrence at one year. B: bilateral; L: left; LL: lower limb; R: right; UL: upper limb. Procedures: C and M were cortical and muscle stimulations.

| **Symptoms** | **Procedure** | **Delay from onset** | **Recovery** |
| --- | --- | --- | --- |
| blindness | C | 4 days | Complete |
| sudden falls | C | 1 weeks | Complete |
| L UL plegia | C | 1 day | Complete |
| L LL plegia | C | 4 days | Complete |
| B LL plegia | C | 5 days | Complete |
| B LL plegia | C | 2 days | Complete |
| B LL plegia | C | hours | Complete |
| gait | C | 2 weeks | Complete |
| gait | C | 1 month | Complete |
| gait | C | 2 weeks | Complete |
| B LL plegia, head tremor | C | 1 month | Complete |
| pseudoballism left lower limb | C | 1 month | Complete |
| tremor | C | years | Complete |
| B LL plegia | C | months | Complete |
| gait ^a^ | C | 1 month | Complete (transient) |
| B UL plegia | C | 1 year | Incomplete |
| R LL plegia | C | 2 months | Incomplete (complete within a day) |
| mutism | C | 1 month | None |
| B LL plegia | C | years | None |
| L LL plegia | C→M | 2 days | Complete |
| B LL plegia | C→M | 1 day | Complete |
| B LL plegia  (recurring brief episodes) | C→M | hours | Complete |
| R LL plegia | C→M | 1 day | Complete |
| B LL plegia | C→M | hours | Complete |
| B LL plegia | C→M | 3 weeks | Complete |
| gait | C→M | 2 months | Complete |
| bilateral recurring hand crispation | C→M | 4 months | Complete |
| R LL+UL tremor | C→M | 5 days | Incomplete |
| R LL plegia | C→M | 5 days | Incomplete |
| R LL plegia | C→M | 2 weeks | Incomplete |
| B LL plegia | C→M | years | Incomplete |
| shaky limb | C→M | 1 month | None |
| gait | C→M | years | None |
| gait, irregular limb tremor | C→M | >2 months | None |
| gait, autobiographic amnesia | C→M | 2 days | None (minor) |
| B LL plegia | M (no C) | hours | Complete |
| R UL plegia | M (no C) | 1 weeks | Complete |
| R LL plegia | M (no C) | hours | Complete |
| gait ^a^ | M (no C) | 1 month | Incomplete |
| L UL plegia | M→C | 1 month | Complete |
| R LL plegia | M→C | 1 year | Incomplete |
